# Personalized Hydration Strategy to Improve Fluid Balance and Intermittent Exercise Performance in the Heat

**DOI:** 10.3390/nu16091341

**Published:** 2024-04-29

**Authors:** Haicheng Li, Kate S. Early, Guangxia Zhang, Pengwei Ma, Haoyan Wang

**Affiliations:** 1 College of Physical Education and Health Sciences, Zhejiang Normal University, Jinhua 321004, China; haicheng0930@163.com (H.L.); zgx981010@163.com (G.Z.); mpw827929278@sina.com (P.M.); 2 Department of Kinesiology & Health Sciences, Columbus State University, Columbus, GA 31907, USA; early_kate@columbusstate.edu

**Keywords:** personalized hydration strategy, fluid balance, sodium loss, intermittent exercise

## Abstract

Sweat rate and electrolyte losses have a large inter-individual variability. A personalized approach to hydration can overcome this issue to meet an individual’s needs. This study aimed to investigate the effects of a personalized hydration strategy (PHS) on fluid balance and intermittent exercise performance. Twelve participants conducted 11 laboratory visits including a VO_2max_ test and two 5-day trial arms under normothermic (NOR) or hyperthermic (HYP) environmental conditions. Each arm began with three days of familiarization exercise followed by two random exercise trials with either a PHS or a control (CON). Then, participants crossed over to the second arm for: NOR+PHS, NOR+CON, HYP+PHS, or HYP+CON. The PHS was prescribed according to the participants’ fluid and sweat sodium losses. CON drank ad libitum of commercially-available electrolyte solution. Exercise trials consisted of two phases: (1) 45 min constant workload; (2) high-intensity intermittent exercise (HIIT) until exhaustion. Fluids were only provided in phase 1. PHS had a significantly greater fluid intake (HYP+PHS: 831.7 ± 166.4 g; NOR+PHS: 734.2 ± 144.9 g) compared to CON (HYP+CON: 369.8 ± 221.7 g; NOR+CON: 272.3 ± 143.0 g), regardless of environmental conditions (*p* < 0.001). HYP+CON produced the lowest sweat sodium concentration (56.2 ± 9.0 mmol/L) compared to other trials (*p* < 0.001). HYP+PHS had a slower elevated thirst perception and a longer HIIT (765 ± 452 s) compared to HYP+CON (548 ± 283 s, *p* = 0.04). Thus, PHS reinforces fluid intake and successfully optimizes hydration status, regardless of environmental conditions. PHS may be or is an important factor in preventing negative physiological consequences during high-intensity exercise in the heat.

## 1. Introduction

Numerous major sporting events take place in the summer season (World Track and Field Championships, Summer Olympic Games, and FIFA World Cup), where players compete in hot environments, often exceeding 30 °C [1]. Exercising in hot and humid environmental conditions can pose additional challenges to physiological function, which can impair aerobic exercise capacity [2]. Heat stress can limit exercise performance in a variety of sports, through many mechanisms including elevated core temperature, rating of perceived exertion, heart rate, and sweat rate [3,4]. In addition, dehydration of greater than 3% of body mass further accelerates the rise in body temperature and increases the risk of exertional heat illness (heat cramps, heat exhaustion or heat stroke) [5,6]. To avoid the excessive loss of body fluids and prevent hindered exercise performance, players require an effective hydration plan pre-, mid-, and post-exercise [7,8].

According to the American College of Sport Medicine (ACSM) hydration guidelines, ~5–10 mL/kg body weight of fluids need to be consumed at least 4 h prior to exercise, and 1.5 L of sodium-containing fluids for each kilogram of body mass loss after exercise [9]. Maintaining water and electrolyte balance during exercise is critical for alleviating heat stress [10]. Typically, players drinking ad libitum does not lead to adequate hydration, particularly during high-intensity exercise [11]. An effective hydration solution that contains sodium can increase fluid retention and compensate for an individual’s fluid loss and sweat sodium loss [12]. Athletics trainers frequently monitor players’ fluid balance and electrolyte losses in daily practice and games, in order to provide an appropriate hydration strategy. However, sweat rates and electrolyte losses can be impacted by the environmental conditions and exercise intensity resulting in a large inter-individual variability. Previous research has shown that sweat sodium losses in sports (soccer, basketball, rugby, etc.) can range from 11.2–86.5 mmol/L [13], with lower sweat rates observed during exercise in cool environments (5 ± 1 °C; range: 0.71–1.77 L/h) compared to hot environments (37 ± 3 °C; range: 1.12–2.09 L/h) [14]. Thus, to prescribe any specific electrolyte solution given the wide variation of fluid and sodium needs for a large population is difficult. To overcome this issue, a personalized approach to hydration, including quantity and electrolyte content, during exercise may better meet each player’s particular needs [15,16]. To our knowledge, no studies have investigated how a personalized hydration strategy affects fluid balance and exercise performance across various environmental conditions, although drinking behavior and thirst perception will be altered by environmental conditions. Therefore, the purpose of the present study was to investigate the effects of a tailored hydration strategy under normothermic and hyperthermic environments on body fluid balance and high-intensity intermittent exercise performance. Researchers hypothesized that a personalized hydration strategy would have positive effects on fluid balance and exercise performance.

## 2. Materials and Methods

### 2.1. Experimental Design

**Figure 1:**
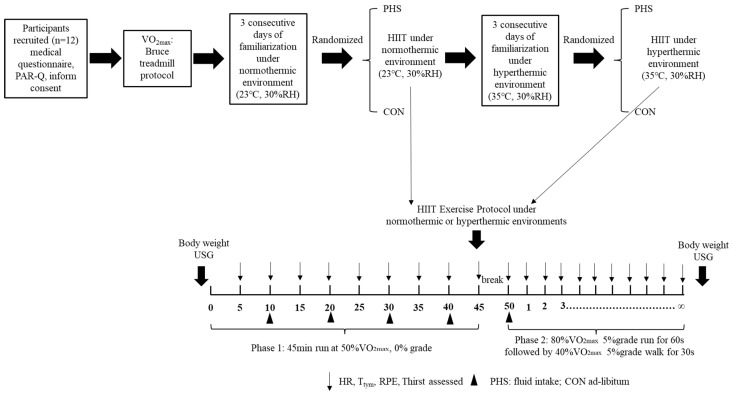
Flow chart of the experimental design and intermittent exercise protocol. PHS: personalized hydration strategy; CON: control group; USG: urine specific gravity; HR: heart rate; T_tym_: tympanic temperature; RPE: rating of perceived exertion.

The present study was a single blinded, randomized cross-over design where each participant completed two, 5-day trial arms in a climate-controlled chamber. The first arm was set as a normothermic environment (NOR: ~23 °C, 30% relative humidity) and the second arm was a hyperthermic environment (HYP: ~35 °C, 30% relative humidity). The experiment was conducted in the spring (April) with the ambient temperature approximately 22–25 °C. As the ambient temperature might influence sweat composition, the environmental conditions of the heated climate-controlled chamber were close to ambient temperature. Participants first completed a maximal oxygen uptake test (VO_2max_; CosMed, K5) using the Bruce treadmill test protocol [17]. The result of VO_2max_ was used to prescribe exercise intensity for all exercise trials. Each arm of the 5-day trial began with three consecutive days of familiarization exercise. Following the three days of familiarization, participants randomly completed a main exercise trial under a control or personalized hydration condition. Participants then crossed over to the second arm with a washout period of seven days (Figure 1). Whole-body sweat rate and sweat electrolyte concentrations were collected during the familiarization exercise phase to prescribe a personalized hydration strategy (PHS). An ad libitum hydration strategy was used as the control group (CON), where the sodium content in the beverage was prescribed as a commercially available electrolyte solution. The four resulting trials were as follows: a normothermic environment with personalized hydration strategy (NOR+PHS), a normothermic environment with ad libitum hydration strategy (NOR+CON), a hyperthermic environment with personalized hydration strategy (HYP+PHS), and a hyperthermic environment with ad libitum hydration strategy (HYP+CON).

### 2.2. Participants

Twelve active, non-acclimatized participants participated in this study, (age: 19.6 ± 0.7 yr; height: 176.6 ± 5.4 cm; weight: 69.5 ± 8.5 kg; maximal oxygen uptake: 53.9 ± 4.4 mL/kg/min). Participants completed a medical history form and a physical activity questionnaire (PAR-Q) before beginning any data collection. All participants were non-smokers and free of any chronic diseases, and sports injuries that would limit their ability to exercise. No alcohol and caffeine were consumed or strenuous exercise performed within 24 h of any exercise trial.

### 2.3. Familiarization Exercise Phase

During the three days of familiarization exercise under both environmental conditions, participants were told to maintain their typical diet. Participants were required to consume 500 mL of fluids 2 h before arriving to ensure a euhydration status. Urine specific gravity (USG) was assessed before exercise with a threshold of <1.020. If USG was greater than 1.020, the trial was rescheduled for the next day. Body mass measures (TANITA HD-395) were taken pre- and post-exercise after participants had voided the bladder. Percent of body mass loss (%BML) was calculated and represented as hypohydration status. The familiarization exercise consisted of running on a treadmill at 50% VO_2max_ for 45 min in the assigned environmental condition. During exercise, participants were provided with plain water drinking ad libitum.

The whole-body sweat rate and electrolyte concentrations were averaged across three days of familiarization exercise for each environmental condition. The whole-body sweat rate was assessed by the differences of body mass pre- and post-exercise corrected by total fluid intake and urine volume. Sweat was collected using an absorbent patch attached to a participant’s lower back [18]. A 70% alcohol spray and dry towel were used to clean the skin surface before adhering the sweat patch to the skin. After exercise, the sweat patch was removed and centrifuged sweat used to analyze electrolyte concentrations (Na^+^, K^+^, Cl^−^; AUDICOM AC9900). In accordance with the analysis of Baker et al., the local sweat sodium concentration was corrected to represent the whole-body loss [19].

### 2.4. Hydration Strategies

To determine the volume of fluid replacement for the PHS during the main exercise trials, the total amount of fluids consumed was equal to sweat volume loss from familiarization exercise. The PHS was made by the lemon-lime Gatorade^®^ Zero electrolyte powder, which contains 230 mg [Na^+^] and 70 mg [K^+^] for every 3 g pack without protein and sugar. As Na^+^ is the main electrolyte in the sweat, Na^+^ concentration in the PHS was prescribed to equal to a participant’s whole-body Na^+^ concentration. In CON, no specific volume of hydration was required and participants were allowed to drink ad libitum. Total fluid intake in CON was the weighed water bottle change from pre- to post-exercise in phase 1. The sodium content of the CON solution was equal to a commercially available electrolyte beverage (~30 mmol/L).

### 2.5. Exercise Trials

During the exercise trial visit, participants were required to consume 500 mL of fluids 2 h before exercise. Participants were told to keep a similar diet and wear the same clothes across exercise trials. Upon arriving to the lab, participants were asked to empty their bladder and give a urine sample for analyzing the USG. If the USG was greater than 1.020, the exercise trial was rescheduled. Body mass was measured and the sweat patch was adhered using the same procedures conducted during the familiarization phase. The exercise trial consisted of two phases with a 5 min break between. The first phase was a 45 min run on the treadmill at 50%VO_2max_, where the hydration strategy was incorporated, and the second phase was a high-intensity intermittent exercise (HIIT) until voluntary fatigue without any fluid provided (Figure 1). In the first phase, participants in the PHS group consumed the total amount of fluid in equally distributed intervals every 10 min, whereas participants in the CON group drank ad libitum. The HIIT protocol was accomplished by running on the treadmill at 80% VO_2max_, 5% grade for 60 s followed by a fast walk at 40% VO_2max_, 5% grade for 30 s until volitional fatigue. The time to exhaustion and bouts completion of HIIT was used to represent the exercise performance across the trials.

### 2.6. Outcome Measures

The heart rate (HR) was measured during all exercise trials (Polar Team-2), and the tympanic temperature (T_tym_; Braun IRT 6030) was assessed to represent body temperature change. In addition, participants were asked to rate perceived exertion (RPE, Borg 6–20) and their perception of thirst. The assessment of thirst perception used a Likert-scale procedure ranging from 0 (no thirst) to 10 (severe thirst). HR, T_tym_, RPE, and thirst perception were assessed every 5 min in the first phase and after each intermittent bout in the second phase.

### 2.7. Statistical Analysis

Statistical analysis was performed using JMPro 16 (SAS Inc.) and data were expressed as mean ± standard deviation (M ± SD). A one-way repeated measure analysis of variance (ANOVA) was used to compare the parameters of fluid balance across trials (NOR+PHS, NOR+CON, HYP+PHS, and HYP+CON). Student-t post-hoc was used for pairwise comparisons between trials. Two-way repeated-measures ANOVA were used to analyze the main outcomes at any specific time point and each bout across trials (trials × time) within 11 bouts of HIIT because no participant completed more than 11 bouts in HYP+CON. The main outcomes included the parameters of fluid balance (%BML, fluid intake, WBSR, LSR, sweat Na^+^, urine Na^+^, and post USG), physiological responses (HR, T_tym_, RPE, and thirst), and exercise performance (time to exhaustion and the number of bouts completed). Statistical significance was accepted at *p* < 0.05.

## 3. Results

### 3.1. Fluid Balance

Table 1 shows the effects of different hydration strategies in the normothermic and hyperthermic environment after exercise trials (phases 1 and 2 averaged) on the parameters of fluid balance. Two participants had a pre-exercise USG greater than 1.020 during the main exercise trial and were rescheduled to the next day. The %BML was significantly lower in NOR+PHS compared to NOR+CON and HYP+CON (all *p* < 0.001). HYP+PHS also had a significantly lower %BML compared to NOR+CON (*p* = 0.002) and HYP+CON (*p* < 0.001). Fluid intake was significantly greater in NOR+PHS (734.2 ± 144.9 g) compared to NOR+CON (272.3 ± 143.0 g; *p* < 0.001) and HYP+CON (369.8 ± 221.7 g; *p* < 0.001). HYP+PHS (831.7 ± 166.4 g) had a significantly greater fluid intake compared to NOR+CON (272.3 ± 143.0 g; *p* < 0.001) and HYP+CON (369.8 ± 221.7 g; *p* < 0.001). Participants in PHS (NOR+PHS: 0.92 ± 0.27 g; HYP+PHS: 1.18 ± 0.29 g) had a significantly greater Na^+^ intake compared to CON (NOR+CON: 0.19 ± 0.10 g; HYP+CON: 0.26 ± 0.15 g, all *p* < 0.001). In addition, HYP+CON had the lowest sweat Na^+^ (56.2 ± 9.0 mmol/L) compared to other trials (*p* < 0.001). HYP+PHS (62.7 ± 9.6 mmol/L) had a significantly lower sweat Na^+^ compared to NOR+PHS (67.8 ± 14.3 mmol/L) and NOR+CON (71.3 ± 16.3 mmol/L, all *p* < 0.001). The changes in urine Na^+^ and urine K^+^ concentrations from pre- to post-exercise were not significant across the trials (all *p* > 0.05). WBSR and LSR were not different between any trials (all *p* > 0.05).

### 3.2. Physiological Responses

Table 2 shows the effects of different hydration strategies under normothermic and hyperthermic environments on physiological responses by exercise phase. In phase 1 when participants ran at a constant workload with fluid accessibility, heart rate was not significantly different across trials (*p* = 0.50). Participants in HYP trials had a significantly higher T_tym_ compared to NOR condition (all *p* < 0.001), and HYP+CON participants had higher T_tym_ (37.1 ± 0.5 °C) compared to HYP+PHS (37.0 ± 0.4 °C, *p* = 0.03). In addition, HYP+CON participants had a significantly greater RPE (10 ± 3) compared to NOR+PHS (9 ± 2, *p* = 0.001), NOR+CON (9 ± 2, *p* = 0.001), and HYP+PHS (9 ± 3, *p* = 0.03). Participants in NOR+PHS had a significantly slower elevated thirst perception (0.5 ± 1.2) in comparison to NOR+CON (0.8 ± 1.0, *p* = 0.004) and HYP+CON (0.7 ± 0.7, *p* = 0.04).

**Figure 2:**
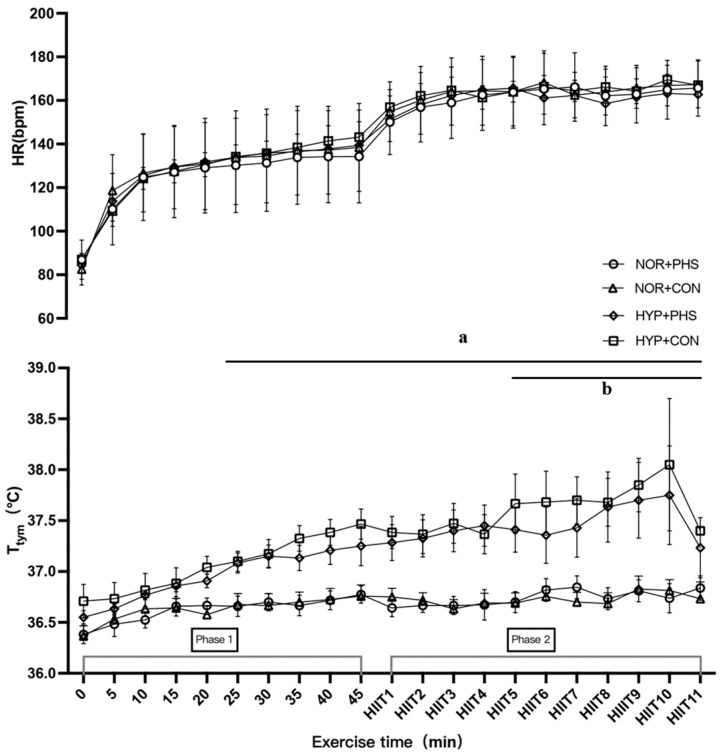
Heart rate (HR) and tympanic temperature (T_tym_) responses during HIIT exercise across groups. NOR: normothermic environment condition; HYP: hyperthermic environment condition; PHS: personalized hydration strategy; CON: drink ad libitum control; ^a^ HYP trials were significantly different to NOR trials, regardless of hydration plan; ^b^ averaged HIIT 5–11 of T_tym_ in HYP+CON was significantly greater compared to HYP+PHS (*p* = 0.02).

**Figure 3:**
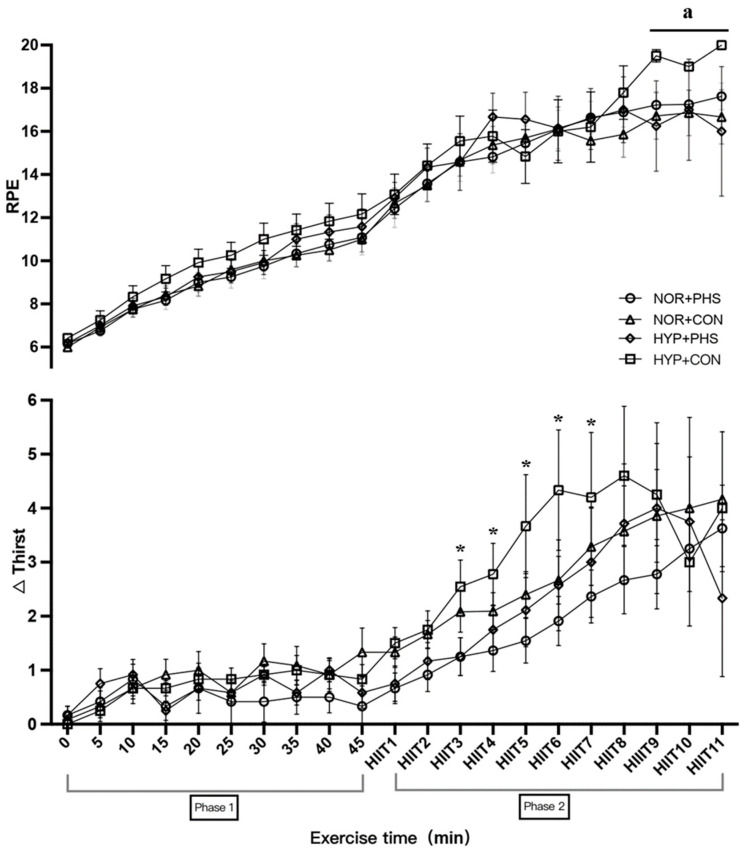
Rating of perceived exertion (RPE) and perception of thirst during HIIT exercise across groups. NOR: normothermic environment condition; HYP: hyperthermic environment condition; PHS: personalized hydration strategy; CON: drink ad libitum control; ^a^ averaged HIIT 9–11 of RPE was significantly greater in HYP+CON compared to other groups (*p* = 0.001). * HYP+CON had significantly greater elevated thirst perception at HIIT3 (*p* = 0.008), HIIT4 (*p* = 0.05), HIIT5 (*p* = 0.04), HIIT6 (*p* = 0.04), and HIIT7 (*p* = 0.04) compared to HYP+PHS.

In phase 2 of HIIT exercise, NOR+CON participants had a greater HR (167 ± 13 bpm) compared to NOR+PHS (163 ± 14 bpm, *p* = 0.006). Higher T_tym_ was found in the HYP trials compared to NOR trials, regardless of the hydration strategy (all *p* < 0.001). However, the two-way ANOVA analysis showed that the averaged T_tym_ from bouts 5–11 of HIIT in HYP+CON (37.7 ± 0.6 °C) was significantly higher compared to HYP+PHS (37.5 ± 0.7 °C, *p* = 0.02; Figure 2). In addition, RPE was not significantly different among trials, but the averaged RPE of HIIT bout 9–11 was significantly greater in HYP+CON (19 ± 1) compared to HYP+PHS (17 ± 3, *p* = 0.005), NOR+CON (17 ± 3, *p* < 0.001), and NOR+PHS (17 ± 2, *p* < 0.001; Figure 3). Participants in HYP+PHS (2.0 ± 2.2) and NOR+PHS (2.2 ± 2.0) had a significantly slower elevated thirst perception compared to NOR+CON (2.8 ± 2.1) and HYP+CON (2.9 ± 2.1, all *p* < 0.01).

### 3.3. Exercise Performance

**Figure 4:**
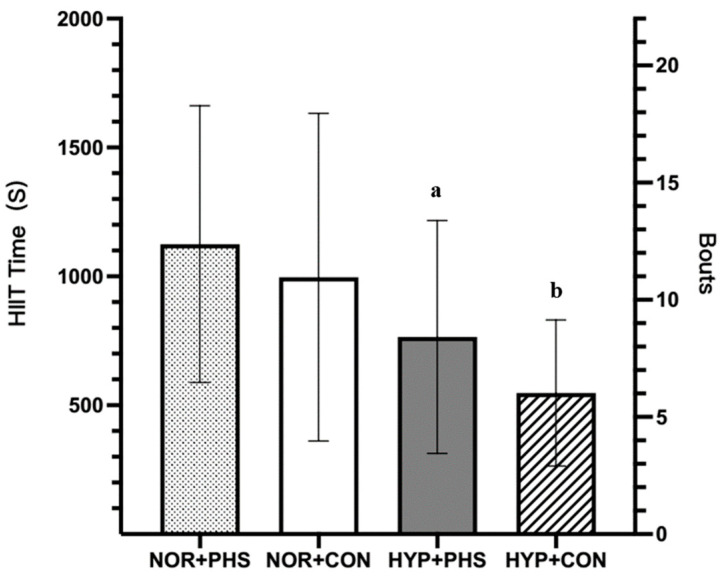
HIIT exercise performance. NOR: normothermic environment condition; HYP: hyperthermic environment condition; PHS: personalized hydration strategy; CON: drink ad libitum control; ^a^ significant difference in HYP+PHS compared to NOR+PHS and NOR+CON (*p* < 0.05); ^b^ significant difference in HYP+CON compared to other trials (*p* < 0.05).

Participants competed 13 ± 6 bouts of HIIT (1125 ± 537 s) under NOR+PHS, 11 ± 7 bouts of HIIT (1005 ± 627 s) under NOR+CON, 9 ± 5 bouts of HIIT (765 ± 452 s) under HYP+PHS, and 6 ± 3 bouts of HIIT (548 ± 283 s) under HYP+CON. Both HYP trials had a significantly shorter HIIT exercise length and less completed bouts compared to NOR trials (HYP+PHS vs. NOR+CON, *p* = 0.03; HYP+PHS vs. NOR+PHS, *p* = 0.002; and HYP+CON vs. NOR+CON, *p* < 0.001; HYP+CON vs. NOR+PHS, *p* < 0.001). In addition, within the HYP condition, HYP+PHS showed a longer exercise length and completion bout compared to HYP+CON (*p* = 0.04). There was no significant difference in HIIT exercise duration between NOR+CON and NOR+PHS (*p* = 0.26; Figure 4).

## 4. Discussion

The main purpose of the present study was to investigate the effects of a personalized hydration strategy under normothermic and hyperthermic environmental conditions on body fluid balance and HIIT exercise performance. The main finding was that participants using a PHS had a significantly greater fluid and sodium intake under both normothermic and hyperthermic environmental conditions, resulting in a blunted hypohydration status. When participants drank ad libitum (control) during the hyperthermic environment, they experienced a greater elevation of thirst perception and RPE during HIIT exercise. Importantly, a PHS under the hyperthermic environment prolonged HIIT exercise performance compared to the CON, suggesting that a targeted approach to hydration can protect performance.

### 4.1. Fluid Balance

Hydration guidelines recommend that players should drink sufficient amounts of sodium-containing fluid to prevent hypohydration and hyperhydration during exercise in the heat. Previous research has reported that drinking ad libitum typically does not lead to successful hydration [20]. The PHS simply derived from exercise losses allowed participants to appropriately hydrate (volume and electrolyte content) without relying on mechanisms like thirst or broad guidelines. Participants also avoided over-hydrating, another problematic issue among players [6]. Emerson et al. prescribed a PHS solution corresponding to ice-hockey players’ sweat volume. Players were required to ingest the PHS solution within an hour of practice, which ultimately resulted in a −0.95% BML, whereas drinking ad libitum resulted in a −1.14% BML [21]. Melo-Marins et al. applied a PHS solution to offset 80% of sweat loss during time-to-exhaustion cycling at 70% of the maximal workload, which resulted in a −0.2% BML compared to ad libitum CON with a −1.0% BML [22]. In addition, Bardis et al. prescribed PHS to offset total sweat loss in a 30-km cycling exercise, which resulted in a −0.5% BML compared to ad libitum a −1.8% BML [23]. Lopez et al. designed a PHS according to a runner’s 4-km sweat loss, multiplying by a factor of five to give the target rehydration volume for a 20-km race, resulting in a −1.4% BML compared to −2.6% BML through ad libitum drinking [24]. Thus, the protection from hypohydration of a PHS described in previous research is consistent with our results. The protocol of PHS in the present study successfully reinforced fluid intake (~700–800 mL) to offset participants’ sweat loss, which resulted in a blunted hypohydration status compared to the ad libitum trials, regardless of environmental conditions. Participants drinking ad libitum replaced nearly 41% of sweat loss, yet still exhibited mild hypohydration status (~−1% BML) under both environmental conditions. Conversely, Lopez et al. found a >2% hypohydration status when drinking ad libitum compared to their PHS, which could be due to the exercise length and intensity being greater than in the present study. Our exercise length was similar to the previous research conducted (Emerson et al. and Bardis et al.) around ~60 min at moderate–vigorous intensity, reflecting similar fluid balance parameters. In contrast to Lopez et al., participants completed a 20-km race in almost 1.7 h with moderate–vigorous exercise intensity. Together, PHS shows a superior effect on hydration status compared to ad libitum drinking during a long bout of high-intensity exercise [19].

Uniquely, the present study prescribed the sodium content in the PHS to compensate for a participant’s sweat sodium loss. Sodium content in a commercially available beverage is typically ~250–270 mg in a 500–600 mL bottle. In the present study, participants required an average of 920 mg and 1180 mg sodium during exercise in the normothermic and hyperthermic conditions, respectively. Participants have to drink 3–4 bottles of commercially available solutions to match their sodium needs, which seems an impractical volume of fluid to drink in such a short time frame. Two previous studies prescribed a sodium content in the PHS [21,25]. Ayotte et al. suggested PHS can provide sodium supplementation in a beverage, especially for those players who engage in prolonged and high-intensity exercise to maintain serum sodium concentration [25]. The sodium content in the PHS of Emerson et al. induced a lowered urine sodium concentration and an increase in potassium from pre- to post-exercise [21]. Similarly, the present study found that urine sodium increased and urine potassium decreased from pre- to post-exercise across all trials, suggesting that participants were in a state of sodium conservation to retain water in the kidney. In addition, sweat sodium concentration exhibited significant differences between PHS and CON under the two environmental conditions. Typically, an individual’s sodium loss is impacted by many factors, such as energy expenditure, heat acclimation, dietary sodium intake, environmental conditions, or a combination of these factors [26]. Sweat sodium concentration is associated with energy expenditure via increased metabolic heat production and sweat rate, consequently the increased rate of sodium excretion in precursor sweat is proportionately greater than the rate of sodium reabsorption [27]. Participants in the normothermic environment had longer exercise duration compared to the hyperthermic environment, which possibly resulted in a greater sweat sodium concentration. On the other hand, when an individual is chronically exposed to a hot environment, an acclimatized state occurs, inducing sweat glands to be more sensitive to aldosterone by lowering sweat sodium concentration. These results corroborate with Bates et al., who also found an individual’s sweat sodium concentration was lower in summer compared to winter [28]. Furthermore, dietary sodium intake affects sweat sodium concentration; higher sodium intake could also result in 10–11% more sweat sodium compared to a low sodium diet [29]. PHS participants consumed greater sodium during exercise, which possibly improves extracellular fluid retention during/after exercise, eliciting a large sweat sodium loss [30]. Therefore, the present study cannot conclude any specific influence factor for the differences in sweat sodium concentration between trials. Future study should focus on the importance of the PHS solution on the sodium loss and water retention of the body.

### 4.2. Exercise Performance

While PHS provides an advantage to optimize a participant’s hydration status and also offset the sodium loss, research studies are consistently looking for a PHS method to improve exercise performance. The present study found that exercise duration was greater in NOR compared to HYP, regardless of hydration strategy, possibly due to environmental stress. Under normothermic environmental conditions, even though participants in the personalized hydration trial had a lower heart rate than in the ad libitum trial, it did not cause any differences in T_tym_ and RPE in the second phase of interval exercise. Consequently, PHS did not show a benefit of exercise performance outcomes. This is similar to Emerson et al., who reported PHS did not have an advantage in exercise performance in the ice-hockey field with a lower environmental stress [21]. Notably, PHS prolonged HIIT exercise duration by nearly 40% compared to CON under hyperthermic environmental conditions. Ayotte et al. and Bardis et al. also suggested that a personalized hydration strategy can improve exercise performance in the heat by improving anaerobic power and heart rate recovery [23,25]. According to the “cardiovascular drift” phenomenon, the combination of hyperthermia and dehydration accelerates the reduction in muscle blood perfusion and exercise performance. Under hyperthermic environmental condition, when participants were allowed to have fluid replacement in the first exercise phase, a PHS attenuates the participant’s T_tym_ and RPE. As they progressed to the second phase of HIIT exercise, even without any fluid intake, PHS participants could still complete more HIIT bouts, finally approaching a similar RPE and T_tym_. In addition, participants drinking ad libitum exhibited 0.2 °C higher T_tym_, averaged from bout 5–11, and they also felt that the exercise was harder at bouts 9, 10, and 11, compared to PHS, and no participant could exercise for more than 11 bouts in the CON trial. This may suggest that a PHS can delay the rise in body temperature and RPE to its peak during exercise in the heat. By contrast, Lopez et al. and Melo-Marins et al. found that a PHS can attenuate the rise in heart rate and skin temperature, but exercise performance remained unchanged. The possible explanation is that Lopez et al. used 4-km fluid loss to estimate a 20-km race fluid replacement, which may not accurately prescribe a PHS. In addition, in the study of Melo-Marins et al., participants were exhausted quickly after exercising for 37–38 min. The hypohydration status may have a small impact on exercise performance, which cannot show the benefits of a PHS. On the other hand, a PHS might blunt the markers of dehydration after a bout of continuous exercise, resulting in better intermittent exercise performance. Notably, under hyperthermic environmental condition, our PHS participants had slower elevation in thirst perception in phase 2 while no fluid was ingested. In the previous study, a high sodium beverage increases thirst perception, like Johannsen et al.’s chicken noodle soup containing 166.9 mmol/L sodium [31]. However, in the present study, the sodium concentration in the PHS is lower than that value (60–80 mmol/L sodium). One of the possible explanations is that participants’ mouths did not dry at the same rate due to the amount of fluid intake in phase 1, leading to less stimulation of the thirst reflex. Hypohydration status typically induced a rise in thirst perception, which is associated with an exercise performance reduction [32,33]. Thus, the present PHS might optimize an individual’s fluid balance and slow elevated thirst perception, which participants might tolerate more in a hypohydration state, and this might aid exercise performance in the heat. This result is so important to those sport events that are significantly affected by hypohydration status and heat stress (outdoor events, heavy sweaters, etc.).

### 4.3. Strengths and Limitations

A strength of the present study was the experimental design with tightly controlled exercise sessions and dietary supervision. The three-day familiarization exercise phase assured that the sweat sodium concentration represented a person’s typical status. In addition, the present study used the regional sweat collecting technique, which is one of the most feasible and popular methods for athletic trainers to assess sweat composition. Considering regional sweat sodium is typically greater than the whole-body sweat sodium concentration, this study corrected the regional sweat sodium concentration to whole-body sweat sodium in accordance with the previous research [19]. Uniquely this cross-over design replicated both normothermic and hyperthermic environmental conditions to compare outcomes. One of the limitations was that we did not measure body weight and analyze electrolyte concentration after phase 1, so that we cannot separate the markers of hydration just like the physiological responses in Table 2. Secondly, there is the possibility that the two beverages used in the study may have different physiological mechanisms with regard to thirst, leading to the PHS group drinking more fluid. Another limitation was that a true control (only water consumed) was not included. However, well-documented research shows a benefit in exercise performance when drinking sodium-containing beverage compared to plain water. So, our study aimed to detect differences in outcomes between PHS and commercially available sports drinks.

### 4.4. Practical Applications

We designed this personalized hydration strategy according to participants’ whole-body sweat rate and sweat sodium concentration that would successfully offset their fluid and sodium loss. Typically, exercise physicians are required to monitor players’ sweat rate and electrolyte losses in daily practice, especially for outdoor team sports. We used absorbent technique to assess local sweat electrolyte concentrations, which is considered one of the most feasible methods to assess a group of players on the field. Exercise physicians can easily prescribe a personalized hydration solution to any athlete. In the normothermic environmental condition, the present results recommend that commercially available electrolyte solutions are sufficient to maintain fluid balance and exercise performance. However, when players compete in the hyperthermic environmental condition or in high-intensity situations, personalized hydration solutions should be prepared to players according to their daily fluid balance assessment.

## 5. Conclusions

A personalized hydration strategy based on an individual’s fluid and sodium loss successfully optimized hydration status, regardless of a hyper- or normothermic environmental condition. In hot environments, a personalized hydration strategy may be more important to improve high-intensity intermittent exercise performance by reducing thirst perception and perception of physical effort. In cool environments, drinking commercially available electrolyte beverages ad libitum is sufficient to maintain exercise performance. Future research should continue investigating a personalized hydration strategy method, considering the least amount of drinking volume necessary to offset sodium loss and maintain a euhydration status.

## Figures and Tables

**Table 1:** The effects of hydration strategies on the parameters of fluid balance (*n* = 12).

	NOR+PHS	NOR+CON	HYP+PHS	HYP+CON	*p* Value
%BML	−0.49 ± 0.40 ^ab^	−1.19 ± 0.57	−0.69 ± 0.40 ^ab^	−1.28 ± 0.34	<0.001
Fluid Intake (g)	734.2 ± 144.9 ^ab^	272.3 ± 143.0	831.7 ± 166.4 ^ab^	369.8 ± 221.7	<0.001
Sodium Intake (g)	0.92 ± 0.27 ^ab^	0.19 ± 0.10	1.18 ± 0.29 ^abc^	0.26 ± 0.15	<0.001
WBSR (L/h)	0.89 ± 0.44	0.89 ± 0.34	1.07 ± 0.20	1.12 ± 0.22	0.16
LSR (mg/cm^2^/min)	3.69 ± 1.10	3.61 ± 1.34	3.86 ± 0.98	4.09 ± 0.80	0.52
Sweat Na^+^ (mmol/L)	67.8 ± 14.3	71.3 ± 16.3	62.7 ± 9.6 ^ab^	56.2 ± 9.0 ^a^	<0.001
ΔUrine Na^+^ (mmol/L)	10.0 ± 17.2	13.9 ± 16.5	5.0 ± 13.6	5.6 ± 16.1	0.36
ΔUrine K^+^ (mmol/L)	−4.3 ± 4.8	−6.0 ± 5.7	−4.8 ± 4.8	−0.5 ± 5.9	0.10

BML—body mass loss; WBSR—whole-body sweat rate; LSR—local sweat rate; NOR—normothermic environment condition; HYP—hyperthermic environment condition; PHS—personalized hydration strategy; CON—drink ad libitum control; ^a^ significant difference compared with NOR+CON; ^b^ significant difference compared with HYP+CON; ^c^ significant difference compared to NOR+PHS.

**Table 2:** The effects of hydration strategies on physiological responses by phases (*n* = 12).

**Phase 1 (0–45 min)**					
	**NOR+PHS**	**NOR+CON**	**HYP+PHS**	**HYP+CON**	***p* Value**
HR (bpm)	124 ± 24	127 ± 24	127 ± 26	127 ± 25	0.50
T_tym_ (°C)	36.6 ± 0.3	36.6 ± 0.4	37.0 ± 0.4 ^abc^	37.1 ± 0.5 ^ab^	<0.001
RPE	9 ± 2 ^c^	9 ± 2 ^c^	9 ± 3 ^c^	10 ± 3	0.003
ΔThirst	0.5 ± 1.2	0.8 ± 1.0 ^a^	0.6 ± 0.8	0.7 ± 0.7 ^a^	0.03
**Phase 2 (HIIT-Fatigue)**					
HR (bpm)	163 ± 14	167 ± 13 ^a^	166 ± 16	165 ± 16	0.04
T_tym_ (°C)	36.8 ± 0.4	36.8 ± 0.3	37.5 ± 0.6 ^ab^	37.6 ± 0.6 ^ab^	<0.001
RPE	16 ± 3	16 ± 3	17 ± 3	16 ± 4	<0.001
ΔThirst	2.2 ± 2.0 ^bc^	2.8 ± 2.1	2.0 ± 2.2 ^bc^	2.9 ± 2.1	0.002
HIIT Time (s)	1125 ± 537	1005 ± 627	765 ± 452 ^abc^	548 ± 283 ^ab^	<0.001
Bouts (#)	13 ± 6	11 ± 7	9 ± 5 ^abc^	6 ± 3 ^ab^	<0.001

HR—heart rate; T_tym_—tympanic temperature; RPE—rating of perceived exertion; NOR—normothermic environment condition; HYP—hyperthermic environment condition; PHS—personalized hydration strategy; CON—drink ad libitum control; ^a^ significant difference compared to NOR+PHS; ^b^ significant difference compared to NOR+CON; ^c^ significant difference compared to HYP+CON; Phase 1: 45 min 50%VO_2max_ run; Phase 2: high-intensity intermittent exercise.

## Data Availability

The data presented in this study are available on request from the corresponding author due to privacy and ethical restrictions.
